# Socioeconomic Determinants of Nutritional Status of Children in Lao PDR: Effects of Household and Community Factors

**DOI:** 10.3329/jhpn.v29i4.8449

**Published:** 2011-08

**Authors:** Yusuke Kamiya

**Affiliations:** Human Development Department Japan International Cooperation Agency 3rd Floor Nibancho Center Building 5-25 Niban-cho Chiyoda-ku Tokyo 102-8012 Japan

**Keywords:** Anthropometry, Child health, Child nutrition disorders, Child nutritional status, Infant health, Infant nutrition disorders, Infant nutritional status, Socioeconomic factors, Laos

## Abstract

The prevalence of undernutrition among Lao children is among the highest in the region. However, the determinants of childhood undernutrition in Laos have not been fully analyzed. This paper, using the dataset of the Lao Multiple Indicator Cluster Survey 3, which is a nationally-representative sample in Laos, investigated the effects of socioeconomic factors at both household and community levels on the nutritional status of children. In the estimation, a multilevel linear model with random-intercepts was used for estimating the determinants of child anthropometric indices. The empirical results revealed that children from households in southern Laos and from ethnic minority groups were less-nourished. Level of education of parents, attitudes of mothers towards domestic violence, assets of household, local health services, and the condition of sanitation and water were considered to be important determinants of nutritional status of children. The pattern of growth-faltering in children by age was identified. Children aged 12-59 months were less-nourished than those aged 0-11 months. The empirical results were consistent with the collective household model which incorporates a decision-making process within the household. Since there is scarce evidence about the predictors of childhood undernutrition in Laos, the findings of this study will serve as a benchmark for future research.

## INTRODUCTION

Global chronic undernutrition in children is highly prevalent and remains a big challenge. One hundred seventy-eight million and 112 million children aged less than five years (under-five children) are stunted (<-2 height-for-age z-scores) and underweight (<-2 weight-for-age z-scores) respectively in low-income countries (1). The Millennium Development Goals (MDGs) address reducing the proportion of underweight children by half between 1990 and 2015. The improvement of childhood nutrition will also assist in the goal to reduce child mortality (MDG 4) because undernutrition is an underlying cause of an estimated more than a half of all deaths of under-five children (2-3). Nutritional status during childhood is important for human development as it affects every phase of human life. Therefore, investment in childhood nutrition contributes not only to improving children's current welfare but to enhancing human's capacity in the long run (4).

Lao People's Democratic Republic (Laos) reduced the rate of under-five mortality from 170 per 1,000 livebirths in 1995 to 75 per 1,000 livebirths in 2006 but the figure is still much worse than neighbouring Thailand (7 deaths per 1,000 livebirths) and Viet Nam (15 deaths per 1,000 livebirths) (Table 1). Besides, the prevalence of childhood undernutrition is also alarmingly high in Laos. During 2000-2007, Laos experienced the worst figure of stunting (40%) and underweight (37%) among under-five children in the Indochina region (5).

Looking at the policies on nutrition, the Government of Laos committed itself to the international obligations of the “Right to Adequate Food” by ratifying the International Covenant on Economic, Social and Cultural Rights in 2007 to ensure that all Lao citizens would be able to avail of their “fundamental right to be free from hunger” (6). In 2008, the Government promulgated the National Nutrition Policy (NNP) to synchronize the international obligations with its national policy [The NNP serves as a “legally binding document to substantially reduce levels of malnutrition, especially vulnerable groups, and to mainstream nutrition in National Socio-Economic Development Plans in line with the implementation of the National Growth and Poverty Eradication Strategy”]. The NNP aims to “accelerate the reduction of malnutrition among all ethnic groups and decrease-associated morbidity and mortality risks” (7).

It has long been recognized that socioeconomic factors, such as poverty, water, sanitation, education, and gender inequality, are important determinants of health outcomes in many low-income countries (8). A proper understanding of the causes of childhood undernutrition assists policy-makers in designing effective strategies which trigger better gains in child health and a further reduction in child mortality. Nonetheless, there exists quite a limited number of studies which investigate the patterns and causes of undernutrition in Laos (9-10). For instance, in 2007, Phengxay *et al*. conducted a cross-sectional study in Luangprabang province to estimate the anthropometric measures for 798 children and investigate their risk factors. They found that low maternal education, poor nutrition knowledge, and restricted intake of meats were the main causes for childhood malnutrition (11). However, to the best of the author's knowledge, there is no empirical study to investigate the socioeconomic determinants of nutritional status of children using a nationally-representative sample in Laos conducted in neighbouring countries, such as Cambodia (12) and Viet Nam (13-14). This study, therefore, aimed at examining the relationship between the socioeconomic factors and the nutritional status of children and also aimed at using the findings as the basis for policy recommendations on nutrition interventions in Laos.

## MATERIALS AND METHODS

The causes of childhood undernutrition are diverse, multidimensional, and interrelated. An analytical framework suggested by the United Nations Children's Fund (UNICEF) encompasses these complexities. It categorizes the causes of childhood malnutrition into (a) immediate causes: inadequate dietary intake and illness, (b) underlying causes: insufficient access to food in a household; inadequate health services and unhealthy environment (poor sanitation and inadequate health services); and inadequate care for children and women at the household level, and (c) basic causes: insufficient actual resources and potential resources at societal level (15-16). Based on this categorization, the causes of childhood malnutrition were divided into variables at the individual, household and community levels for analysis.

### Econometric model

To estimate the determinants of nutritional status of children, the study applied a reduced-form equation derived from the standard Beckerian household utility function of consumer demand (17) and Grossman's health production function (18). A multilevel linear model was particularly applied considering heterogeneities at both mother and community levels. It is based on the assumption that the nutritional status of children born to the same mother is not independent because of unobservable factors, such as biological inheritance. Similarly, unobserved community characteristics, such as local climate, infectious diseases, and food availability, may affect children's nutrition. Consequently, the model was expressed as:

CHILDH_IJK_ = α + X’ _ijk_ β + u_j_ + u_k_ + ϵ_ijk_ [1]

where CHILDH_IJK_ is a vector of anthropometric measures of a child born to a mother *j* in a community *k;*

X’ _ijk_ is a vector of covariates at the child, household and community levels;

u_j_ and u_k_ refer to an unobserved mother-specific random effect and a community-specific random effect respectively; and

ϵ_ijk_ is an error component.

To test the validity of the collective household model, which takes into account a decision-making process within the household (19), the household covariates included the educational attainment by both mother and father and also information on domestic violence.

### Data and descriptive statistics

The dataset used for the study was from the Lao Multiple Indicator Cluster Survey (MICS) 3 collected in 2006. The Lao MICS 3 includes demographic, socioeconomic and health information from a nationally-representative sample in Laos. The main sampling domains were three regions—North, Central, and South of Laos. Within each region, 100 clusters were selected with probability proportional to size. Then, 20 households were systematically sampled from each cluster. Accordingly, the sample was designed to collect information about 6,000 households in total (3 regions × 100 clusters × 20 households). Finally, 5,894 households—comprising 33,100 members (including 4,204 under-five children)—were successfully interviewed (20). For analysis, 411 cases were excluded from the original sample of 4,204 children because they lacked information about at least one explanatory variable used for regression analysis. Consequently, the sample was limited to 3,793 children aged 0-59 months born to 2,970 mothers in 300 communities (=3 regions × 100 clusters).

**Table 1.** Socioeconomic and health indicators in the Indochina region (5)

Indicator	Laos	Cambodia	Myanmar	Thailand	Viet Nam
Gross national income per capita (US$) (2007)	540	580	220	3,400	790
Under-5 mortality rate (2007)	70	91	103	7	15
% of stunting among under-5 children (2000-2007)*	40	37	32	12	36
% of underweight among under-5 children (2000-2007)*	37	36	32	9	20
% of population using improved drinking-water sources (2006)	65	60	80	98	66
% of population using improved sanitation facilities (2006)	28	48	82	96	46

* According to the definition by the National Center for Health Statistics/Centers for Disease Control and Prevention/World Health Organization reference (21)

To assess the nutritional status of children, three anthropometric z-scores based on the World Health Organization/Centers for Disease Control and Prevention/National Center for Health Statistics reference were used (21). Height-for-age z-score is a longer-term index which represents linear growth of a child. It gives information about chronic undernutrition or ‘stunting’ which reflects the accumulation of past outcomes. Weight-for-age z-score is an index of both acute and chronic undernutrition which provides information about ‘underweight’. Weight-for-height z-score is a shorter-time index which indicates acute undernutrition or ‘wasting’. Wasting is usually caused by a recent nutritional deficiency and may manifest significant seasonal variations according to changes in the availability of food or prevalence of disease. A child whose height-for-age, weight-for-age, or weight-for-height is more than 2 standard deviations below the median of the reference population is classified as moderately or severely stunted, underweight, and wasted respectively. Those whose height-for-age, weight-for-age, or weight-for-height are more than 3 standard deviations below the median are classified as severely stunted, underweight, and wasted respectively. Table 2 shows the prevalence of undernutrition among the sample children aged 0-59 months, measured by z-scores. There are large variations in childhood undernutrition across age-groups, regions, and ethnic groups.

The figure presents the changes in the mean z-scores by age for children aged 0-59 months, stratified by three-month age bracket. The overall patterns of the z-scores were similar to the observations from 39 developing countries by Shrimpton *et al*. (22). The weight-for-age scores were more close to zero than the other indices. Both weight-for-age and height-for-age declined steeply just before the 24th month, which is considered a critical age of child growth (4,23), stabilizing at around −2.0. Thereafter, weight-for-age recovered a little and kept its score between −2.0 and −1.5 until the 60th month of age. Height-for-age fluctuated more than the other indices within the range of −2.5 to −1.5.

Table 3 displays the mean values of z-scores by community. It was confirmed that there was a large gap in the nutritional status of children between the best and the worst communities for each of the z-scores. For instance, the mean height-for-age z-scores varied from −2.9585 in the worst to 0.9483 in the best community. The wide variations of z-scores among communities imply that there are large heterogeneities at the community level to affect the nutritional status of children.

Table 4 shows descriptive statistics for regression analysis. The data were corrected for a cluster survey design by calculating sampling weights which were available in the MICS 3 dataset. The child variables consisted of sex and age. Slightly more than a half (51.3%) of the sample children were male. The age of the child was captured by the dummy variables in 12-month bracket during 0-59 months (with the reference group of 0-11 months of age). The household variables comprised information about ethnicity (Lao, Khmou, Hmong, and other ethnic language groups), age of mother, educational achievement by mother and father, attitude of mother towards domestic violence and number of children, wealth index score, and regions (Central, North, and South). Results of studies confirmed that the relative status of women compared to men within the household had a positive impact on child health through women's stronger position in decision-making in labour supply, allocation of income, provision of childcare, and healthcare-seeking behaviours (24-26). In the Lao MICS 3, several questions were asked about women to assess their attitudes towards domestic violence. A dummy variable was created of whether a woman agrees that a husband is justified in hitting or beating his wife for at least one of the following reasons: (a) when she goes out without telling him; (b) when she neglects children; (c) when she argues with him; (d) when she refuses sex with him; and (e) when she burns food. This dummy variable served as a proxy for domestic violence against wives by their husbands, assuming that women who agreed with justification for husband's violence under the above situations were more likely to be abused by their husbands in their real life (20). Eighty-five of the sample mothers believed that the above reasons provided justification for beating. The last household variable was an asset index score which was included as a proxy for income of the non-labour household. The score was calculated for each household using principal component analysis, and it was readily available in the MICS 3 dataset.

**Table 2.** Prevalence of undernutrition* among children aged 0-59 months in Lao PDR

Prevalence of undernutrition	Height-for-age		Weight-for-age		Weight-for-height	
	Severely or moderately stunted (<-2 SD)	Severely stunted (<-3 SD)	Severely or moderately underweight (<-2 SD)	Severely underweight (<-3 SD)	Severely or moderately wasted (<-2 SD)	Severely wasted (<-3 SD)
Sex (%)						
Male	39.3	15.3	36.0	9.1	7.2	1.0
Female	39.9	16.7	36.8	9.7	5.9	0.8
Age (months) (%)						
0-11	16.8	5.2	12.3	3.5	4.2	0.6
12-23	42.2	14.8	44.3	12.1	12.8	0.8
24-35	41.3	17.0	44.6	12.8	7.8	1.5
36-47	46.8	21.9	41.4	9.6	3.3	0.8
48-59	50.5	20.7	38.2	8.4	4.9	0.9
Region (%)						
Central	33.7	12.1	32.1	7.5	6.5	1.1
North	42.5	18.4	33.0	7.8	4.1	0.8
South	45.8	19.4	49.0	15.0	10.2	0.6
Ethnicity (%)						
Lao	30.1	10.5	32.4	7.3	7.0	0.9
Khmou	47.5	20.0	36.9	9.5	3.4	0.7
Hmong	46.4	18.7	27.4	4.3	2.4	0.3
Others	50.8	23.7	51.2	17.5	10.4	1.6
Total	**39.6**	**16.0**	**36.4**	**9.4**	**6.6**	**0.9**

* According to the definition by the National Center for Health Statistics/Centers for Disease Control and Prevention/World Health Organization reference (21);

SD=Standard deviation

**Fig.**
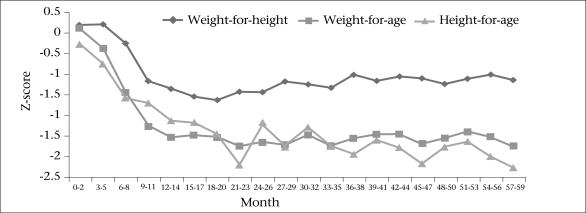
Mean of z-scores by 3-month age-groups during 0-59 months

**Table 3.** Mean of anthropometric z-scores by community

Anthropometric z-score	Observations	Mean	Standard deviation	Minimum	Maximum
Height-for-age	300	-1.5624	0.6819	-2.9585	0.9483
Weight-for-age	300	-1.5644	0.5426	-2.8500	0.6200
Height-for-weight	300	-0.7763	0.4745	-2.8175	1.5950

**Table 4.** Descriptive statistics

Variable	No.	Mean	Standard deviation	Minimum	Maximum
Child variables					
Height-for-age z-score	3,791	-1.7068	1.5754	-9.05	9.62
Weight-for-age z-score	3,793	-1.6180	1.1850	-5.91	7.93
Weight-for-height z-score	3,765	-0.7409	0.9813	-6.29	7.51
Male: age (months)	3,793	0.5134	0.4999	0	1
0-11	3,793	0.2001	0.4001	0	1
12-23	3,793	0.2022	0.4017	0	1
24-35	3,793	0.2023	0.4017	0	1
36-47	3,793	0.2248	0.4175	0	1
48-59	3,793	0.1706	0.3762	0	1
Household variables					
Ethnic group					
Others	3,793	0.2281	0.4196	0	1
Lao	3,793	0.4722	0.4993	0	1
Khmou	3,793	0.1452	0.3524	0	1
Hmong	3,793	0.1532	0.3602	0	1
Age of mother	3,793	28.6634	6.8895	15	48
Education of mother	3,793	0.4051	0.4910	0	1
No education					
Primary	3,793	0.4484	0.4974	0	1
Secondary	3,793	0.1466	0.3537	0	1
Education of father					
No education	3,561	0.1949	0.3962	0	1
Primary	3,561	0.4915	0.5000	0	1
Secondary	3,561	0.3136	0.4640	0	1
Domestic violence	3,793	0.8520	0.3552	0	1
No. of children per household	3,793	1.7295	0.8227	1	7
Wealth index score	3,793	-0.3314	0.8391	-1.36832	3.109071
Region					
Central	3,793	0.4142	0.4927	0	1
North	3,793	0.3463	0.4759	0	1
South	3,793	0.2394	0.4268	0	1
Community variables					
Use of antenatal care*	3,793	0.3356	0.3435	0	1
Use of skilled birth attendance*	3,793	0.1801	0.2952	0	1
Child vaccination, all types†	3,793	0.2236	0.2134	0	1
Vitamin A for child†	3,793	0.3675	0.2314	0	0.9473684
Use of bednet the night before†	3,793	0.8679	0.2024	0	1
Incidence of childhood diarrhoea‡	3,793	0.1266	0.1092	0	0.5238096
Possession of a latrine¶	3,793	0.3702	0.3873	0	1
Time to get water (minutes)¶	3,793	9.1158	8.9281	0	52.7
Possession of radio/TV¶	3,793	0.6061	0.2339	0.05	1

* The proportion of children per community where mothers used these services at their last birth-delivery;

† The proportion of children per community, who received or used these services;

‡ The proportion of children per community, who had diarrhoea in the two weeks preceding;

¶ The proportion of households per community; TV=Television

The community variables included the characteristics which represented the local health system, sanitation, water, and communication infrastructure in each community. The local health system variables included: the proportion of children per community where mothers received antenatal care by skilled personnel during the last pregnancy; the proportion of children per community where mothers gave birth with assistance of skilled personnel at the last delivery; the proportion of children per community, who received all the recommended vaccines—BCG, DPT, polio, and measles; the proportion of children per community, who received vitamin A supplement; and the proportion of children per community, who slept under an insecticide-treated net in the previous night. The water and sanitation variables included the proportion of households per community which had a latrine, households’ average time per community to get water, and the proportion of children per community, who had diarrhoea in the preceding two weeks. The level of the communication infrastructure, which measures the accessibility to information about child health improvement, was proxied by the proportion of households per community, which owned a coloured television and/or a radio.

## RESULTS

Table 5 presents the estimated coefficients and p values for children aged 0-59 months for the three anthropometric z-scores. In the estimation, a multilevel linear model with random-intercepts expressed in equation [1] was performed using the Stata 10 ‘xtmixed’ command (27). As to the choice of model, the results of likelihood-ratio (LR) tests (χ^2^: LR test vs linear regression) suggest that a multilevel linear model with random-intercepts is preferable to an ordinary linear model with fixed-intercept in the estimation for all the three z-scores (p≤0.001 for height-for-age, weight-for-age, and weight-for-height). Looking at the results of child's characteristics, there was no significant difference between male and female children for any of the z-scores. As expected, the age of the child was significantly associated with undernutrition as measured by all the three z-scores in a cumulative manner. Children aged 12-59 months were much more undernourished than those aged less than 12 months (p<0.01). Noticeably, the size of an estimated coefficient suggests that the height-for-age z-score kept falling throughout all the age-bracket until 48-59 months of age. In contrast, the weight-for-age and weight-for-height z-scores decreased up to 24-35 months and 12-23 months of age respectively and levelled off thereafter.

### Household characteristics

Children from ethnic Lao group were more nourished than the reference population (the other ethnic groups) in terms of all the three anthropometrics. Children from Khmou group had higher weight-for-height, and children from Hmong group had higher weight-for-age and weight-for-height scores than the reference population. Children from the other ethnic groups were the least nourished even after controlling for the socioeconomic factors, for which the population was disadvantageous. It implies that the ethnic minority groups in Laos are more disadvantageous in terms of children's welfare, in addition to the economic condition (28).

**Table 5.** Results of estimations

Variable	Height-for-age		Weight-for-age		Weight-for-height	
	Coefficient	p value	Coefficient	p value	Coefficient	p value
Child variables						
Male: age† (months)						
0-11	0.0078	0.8670	0.0036	0.9160	-0.0420	0.1480
12-23	-0.9947***	0.0000	-1.1629***	0.0000	-0.9043***	0.0000
24-35	-1.0363***	0.0000	-1.2180***	0.0000	-0.7716***	0.0000
36-47	-1.2967***	0.0000	-1.1349***	0.0000	-0.6136***	0.0000
48-59	-1.3366***	0.0000	-1.1171***	0.0000	-0.6089***	0.0000
Household variables						
Ethnic group					
Others†						
Lao	0.1873**	0.0130	0.1548***	0.0090	0.1056**	0.0490
Khmou	-0.1234	0.2310	0.0839	0.3030	0.2320***	0.0020
Hmong	0.0119	0.9040	0.4108***	0.0000	0.5299***	0.0000
Age of mother	0.0675**	0.0130	0.0229	0.2470	-0.0222	0.1900
Square of age of mother	-0.0010**	0.0230	-0.0003	0.3100	0.0003	0.2290
Education of mother						
No education†						
Primary	0.0397	0.5240	0.0433	0.3470	0.0363	0.3570
Secondary	0.0719	0.4780	0.0931	0.2110	0.0964	0.1300
Education of father						
No education†						
Primary	0.1873***	0.0080	0.1397***	0.0070	0.0561	0.2050
Secondary	0.2387***	0.0060	0.1922***	0.0030	0.0852	0.1180
Domestic violence	-0.1537**	0.0320	-0.1414***	0.0070	-0.0395	0.3800
No. of children per household	-0.0166	0.6320	-0.0023	0.9280	0.0203	0.3500
Wealth index score	0.2034***	0.0000	0.0978**	0.0110	-0.0213	0.5220
Region
Central†						
North	-0.0684	0.3910	0.0315	0.6240	0.0772	0.1960
South	-0.2939***	0.0000	-0.3116***	0.0000	-0.2198***	0.0000
Community variables						
Use of antenatal care	-0.1179	0.2780	-0.1544*	0.0770	-0.1253	0.1240
Use of skilled birth attendance	-0.0246	0.8600	0.1595	0.1430	0.1860*	0.0620
Child vaccination, all types	0.0165	0.9140	-0.0161	0.8950	-0.0215	0.8490
Vitamin A for child	0.1201	0.3390	0.1901*	0.0610	0.1352	0.1550
Use of bednet the night before	0.3162**	0.0350	0.0197	0.8740	-0.1788	0.1280
Incidence of childhood diarrhoea	-0.3937*	0.0970	-0.2927	0.1300	-0.0401	0.8250
Possession of a latrine	0.1635*	0.0740	0.1065	0.1520	0.0300	0.6690
Time (minutes) to get water	-0.0054	0.1190	-0.0065**	0.0210	-0.0044*	0.0980
Possession of radio/TV	0.2098	0.1510	0.1379	0.2460	-0.0160	0.8860
Constant	-2.1702***	0.0000	-1.2498***	0.0000	0.1409	0.6280
Wald χ^2^ (29)	710.31***	0.0000	1101.9***	0.0000	713.79***	0.0000
χ^2^: LR test vs linear regression	20.49***	0.0000	37.03***	0.0000	45.36***	0.0000
Community random effect (SD)	0.0293		0.1438		0.1796	
Mother's random effect (SD)	0.5186		0.4134		0.3148	
Sample-size (no. of children)	3,559		3,561		3,534	
No. of communities	280		280		280	
No. of mothers	2,565		2,567		2,551	

*p<0.1;

** p<0.05;

*** p<0.01;

† Reference group;

LR=-Likelihood ratio;

SD=Standard deviation;

TV=Television

Educational attainment of mothers did not exert any positive impact on childhood nutrition, contrary to numerous previous studies, when it was estimated together with education of fathers. On the other hand, both primary and secondary schooling of fathers positively correlated with children's height and weight-for-age. The insignificance of mother's educational effect might be caused by the correlation between education of mothers and education of fathers. To scrutinize this point, a correlation coefficient was first calculated between the categorical variables of education of mothers and education of fathers. Correlation was high for secondary education (0.4451) while it was low for primary education (0.1649). Next, the effects of education of mothers and fathers on children's nutrition were estimated in a separate regression equation. Table 6 summarizes the results and shows that primary and secondary education of mothers had significant and positive impacts on child's height-for-age, albeit with a small statistical significance (0.05<p<0.1). Secondary education of mothers also positively correlated with children's weight-for-age. Nevertheless, education of fathers had a stronger and broader effect on children's nutrition, with larger coefficients for all the z-scores and a significant impact on weight-for-height. The dummy variable representing a domestic violence had significant and negative effects on both height and weight-for-age. These results indicate that the collective household model is more acceptable than the unitary household model. With regard to the regional differences, children from households located in southern Laos were less-nourished than those from the central region for all the z-scores.

**Table 6.** Education effects by mother and father when they are estimated separately

Variable	Height-for-age		Weight-for-age		Weight-for-height	
	Coefficient	p value	Coefficient	p value	Coefficient	p value
Education of mother†						
Primary	0.0964*	0.0990	0.0697	0.1060	0.0342	0.3530
Secondary	0.1616*	0.0870	0.1404**	0.0430	0.0931	0.1170
Education of father†						
Primary	0.1976***	0.0040	0.1502***	0.0030	0.0644	0.1370
Secondary	0.2577***	0.0020	0.2134***	0.0010	0.1045**	0.0480

†Reference group is ‘No education’;

*p<0.1;

**p<0.05;

***p<0.01

### Community characteristics

The variables representing the local health systems exhibited some complex results. Contrary to the expectation, the proportion of children per community whose mothers received antenatal care from skilled personnel during the last pregnancy exhibited a negative impact on weight-for-age. It might be caused by the opposite causality, i.e. mothers whose children were less-weighted were more likely to seek antenatal care once they became pregnant to prepare themselves for their next delivery. On the other hand, the proportion of children whose mothers were attended by skilled personnel at the last delivery positively correlated with weight-for-height but not with the other two z-scores. The variables representing the coverage of vitamin A supplementation and the bednet were both positively associated with weight-for-age. All the variables reflecting sanitation and water exerted the expected results—the prevalence of childhood diarrhoea had a negative impact on height-for-age, the latrine coverage positively correlated with height-for-age, and households’ average time to get water had negative impacts on weight-for-age and weight-for-height. The coverage of radio and/or television, which represented the communication infrastructure providing information about child health improvement, was not statistically significant for all the z-scores.

## DISCUSSION

This study has provided evidence on the socioeconomic determinants of child health outcomes in Laos. Some important and relevant policy implications are drawn from the empirical findings. There is now a global consensus that certain types of nutrition interventions, such as breastfeeding, supplementation of micronutrients, and some healthcare services are effective in improving the nutritional status of children in low-income countries (29-30). These interventions are primarily targeted for eliminating immediate causes of childhood undernutrition at the individual level. The empirical results indicate that the causes of undernutrition among Lao children are rooted in socioeconomic factors. It implies that shorter-term interventions which aim to remove the immediate causes are not sufficient to achieve longer-term improvement in child health but should be undertaken together with broader social policies to tackle the underlying and basic causes. For example, the empirical results showed that the poor condition of sanitation and water had negative impacts on both acute and chronic undernutrition of children. Since the percentage of population with access to improved sanitation facilities in Laos is the lowest (28%) in the region (Table 1)., investment in sanitation facilities should be given a high priority as a country strategy for social and human development.

The results have also provided evidence on the socioeconomic background of undernourished children in Laos. Children from the disadvantaged households, in terms of geographical location, ethnicity, parental education, household's asset, availability of local health services, sanitation, and water, suffered a greater risk of being undernourished than those living in the better-off environment. Considering the limited financial and human resources for health spending in Laos, priority of social policies for improving nutrition should be given to the most vulnerable children. Consequently, policy-makers should deliberate a plan on how to deliver necessary resources to such populations by collecting more evidences on the profile of undernourished children in Laos.

There are some caveats to be considered when interpreting the results. First, the direction of causality from the socioeconomic factors to the child health outcomes is not fully confirmed due to the nature of cross-sectional data. Nonetheless, the causality would be plausible if there were no omitted variables in the estimation models. Another point is how to interpret the differential impacts between schooling of mothers and schooling of fathers on nutrition of children. Since the Lao MICS 3 does not contain detailed information about education of adults, further investigation using other datasets will be required. Despite the limitations, the study is the first one to measure the socioeconomic determinants of nutritional status of children in Laos and will, therefore, serve as a benchmark for further studies.

## ACKNOWLEDGEMENTS

The author thanks the anonymous referees of this journal and Dr. Kara Hanson, London School of Hygiene & Tropical Medicine, for their insightful comments and suggestions.
